# Longitudinal assessment of S100B serum levels and clinical factors in youth patients with mood disorders

**DOI:** 10.1038/s41598-021-91577-6

**Published:** 2021-06-07

**Authors:** Aleksandra Rajewska-Rager, Monika Dmitrzak-Weglarz, Pawel Kapelski, Natalia Lepczynska, Joanna Pawlak, Joanna Twarowska-Hauser, Maria Skibinska

**Affiliations:** 1 Department of Psychiatric Genetics, Chair of Psychiatry, Poznan University of Medical Sciences, Rokietnicka 8, 60-806 Poznan, Poland; 2 Department of Child and Adolescent Psychiatry, Karol Jonscher Clinical Hospital, Poznan University of Medical Sciences, Szpitalna 27/33 St, 60-572 Poznań, Poland

**Keywords:** Biomarkers, Diseases of the nervous system

## Abstract

Mood disorders have been discussed as being in relation to glial pathology. S100B is a calcium-binding protein, and a marker of glial dysfunctions. Although alterations in the S100B expression may play a role in various central nervous system diseases, there are no studies on the potential role of S100B in mood disorders in adolescents and young adults . In a prospective two-year follow-up study, peripheral levels of S100B were investigated in 79 adolescent/young adult patients (aged 14–24 years), diagnosed with mood disorders and compared with 31 healthy control subjects. A comprehensive clinical interview was conducted which focused on clinical symptoms and diagnosis change. The diagnosis was established and verified at each control visit. Serum S100B concentrations were determined. We detected: lower S100B levels in medicated patients, compared with those who were drug-free, and healthy controls; higher S100B levels in a depressed group with a family history of affective disorder; correlations between age and medication status; sex-dependent differences in S100B levels; and lack a of correlation between the severity of depressive or hypo/manic symptoms. The results of our study indicate that S100B might be a trait-dependent rather than a state-dependent marker. Due to the lack of such studies in the youth population, further research should be performed. A relatively small sample size, a lack of exact age-matched control group, a high drop-out rate.

## Introduction

Mood disorders are associated with significant psychosocial impairment marked by changes in mood, energy, thinking, and behavior. The onset of major depression (MDD) or bipolar disorder (BD) may appear gradually or suddenly during childhood, adolescence, or adulthood. Retrospective studies show that 50–66% of adult patients diagnosed with bipolar disorder reported illness onset before age 18 and 15–28% before 13 years old^1^. During adolescence, development and growth of brain structures with construction and strengthening of nerve circuits and pathways are observed. Specific parts of the brain may mature at different rates and times, and it is estimated that brain maturation is incomplete until an individual is around 25 years old^2,3^. This has an important implication for the self-regulation process in this age, which plays an essential role in the management of controlling behavior emotions and motivation^4^. Consequently, in relation to the brain maturation process, the symptoms and diagnosis of psychiatric illness may change as the child grows. While the familial risk of affective disorders is well established, potential clinical or biomarkers for later illness development in youths remain unknown. Many studies indicate that neurotrophic factors are associated with neuropsychiatric disorders, including mood disorders. Brain-derived neurotrophic factor (BDNF) affects the differentiation of neurons in early development, also affecting synaptic plasticity and neuronal survival in adulthood. Due to the postulated glial hypothesis of affective disorders and brain neuroplasticity, there has been a growing interest in a calcium-binding protein (S100B) and its possible role in mood disorders^5^. Disturbances in the blood–brain barrier permeability may be one of the factors that predispose to the occurrence of mood disorders, and being a useful biomarker of the changes in glial tissue, the S100B protein was considered. This protein is mostly derived from glial cells, and plays a part in the regulation of the metabolism of neural cells. It enhances the survival of neurons and supports the development of serotoninergic neurons. Depending on its concentration, it has different effects on nerve cells. Calcium-binding protein has a protective effect on the nerves and plays a role in nerve cell development and regeneration if it is kept within normal physiological levels (nanomolar range). Studies performed after neurological injuries have shown that extracellular S100B in micromolar concentrations can cause apoptosis and neurodegeneration and stimulate the expression of proinflammatory cytokines^6,7^. Previous studies assessing the effects of S100B on serotonergic regulation in animals show that S100B affects the survival of neurons by increasing it during development and that S100B can be regulated by serotonin receptors^8^. Decreased S100B mRNA expression in amygdala, hippocampus, hypothalamus, prefrontal cortex, and striatum of a genetic rat model (Flinders Sensitive Line) for depression was reported^9^. These results are partly in line with a study on a rat chronic unpredictable stress (CUS) model, where lower mRNA S100B expression was found in the prefrontal cortex, and no differences in the striatum and hippocampus^10^. In the study by Ye et al. (2011) opposite results were obtained, with increased expression of S100B mRNA in the hippocampus of CUS rats^11^.

In humans, the gene encoding S100B protein is located on the 21q22.3 chromosome. This region is also considered to play a significant role in bipolar disorder^12^. Only a few genetic association studies on *S100B* gene polymorphisms were conducted in mood disorders. In a Chinese population two single nucleotide polymorphisms were studied and no association with major depressive disorder was detected, although rs9722 was correlated with the age of onset^13,14^. Association of rs3788266 with psychotic subtype of bipolar disorder in the Caucasian population was reported^12^. Dagdan et al. (2011) demonstrated that the G allele of rs3788266, polymorphism located within the promoter region of *S100B* gene is associated with an increased S100B serum protein level in both, bipolar disorder patients and healthy controls in the Irish and German population^15^.

Clinical studies in adult patients indicate that S100B is elevated in mood disorders and might be a potential biomarker for mood disorders and successful antidepressive treatment. The meta-analysis showed the increased level of S100B protein in blood serum and cerebrospinal fluid of both patients with depressive disorders and bipolar disorder, as compared to control groups^16^. Despite the existence of several studies on S100B in adult patients, there are still no such studies in the younger group. Based on the kindling hypothesis, researches over biological mechanisms of affective disorder in adolescents and young adults brings us closer to better understanding the pathophysiology of this disorder. Our study explores if serum S100B levels differ between healthy controls and youths depressed or bipolar patients. Comparing these three patients groups (controls, depressive, bipolar) during a different time point and symptoms severity (acute episodes and then reaching euthymia) may help us explore the role of altered S100B levels during illness continuity. To do so, primary we investigated a possible correlation between the serum levels of S100B and study subjects. Correlation of serum S100B with other variables such as clinical parameters (age, gender, family history, diagnosis change) was considered as secondary outcomes.

### Hypotheses

According to our knowledge, this is the first prospective study that tries to explore the correlation between S100B serum levels and mood disorders in adolescents and young patients. Based on the findings on glial pathology in mood disorders in adult patients^17,18^ in our study, we provided some research questions following our hypothesis: (1) there are any differences in baseline serum S100B levels between depressed, hypomanic patients and healthy controls (2) could baseline S100B levels be influenced by clinical factors (severity of depressive and manic symptoms, medication status, family history of psychiatric and affective disorders, or gender) (3) is there a change between baseline S100B concentrations and euthymic state (as well as in a 2-year follow-up) and finally (4) do different baseline S100B levels predict diagnosis change from unipolar to bipolar disorder in young patients.

## Methods

### Settings and study group

The study took place in the Psychiatric Department of University of Medical Sciences in Poznan, Poland. Between 2012 and 2017 we included eighty patients aged 14–24 in a 2 year prospective study, each with a diagnosis of mood disorders: unipolar disorder or bipolar disorder. The Ethics Committee approved the study protocol at the University of Medical Sciences, Poznan (no. 362/11). The study was performed in accordance with the Declaration of Helsinki.

Sixty patients (mean age 17.6 (SD 2.92), 48 females, 12 males) were recruited from in-patient wards: the Child and Adolescent Psychiatry Unit and the Adult Psychiatry Unit from the Department of Psychiatry at the University of Medical Sciences in Poznan, as well as nineteen patients (mean age 21.6 (SD 2.43), 8 females, 11 males) from outpatient departments. Healthy controls were drawn from university students and secondary school students all volunteers from Poznan. The control group consisted of 31 healthy persons, mean age 21.15 (SD 2.68, female n = 26, male = 5) without psychiatric symptoms and no history of psychiatric disorders, substance abuse, or severe medical problems. The demographic data are presented in Table 1.

**Table 1:** Characteristics of the study group at baseline.

	All patients	Depressed	Hipo/manic	Mixed episodes	Control group
Total count	79	52	17	10	31
Female/male	56 /23	39 /13	9/8	8 /2	26/5
Mean age	18.59 (± 3.28)	18.67 (± 3.54)	18.88 (± 2.93)	17.70 (± 2.45)	21.15 (± 2.68)
Mean age at illness onset	16.77 (± 2.76)	16.82 (± 2.96)	17.00 (± 2.63)	16.20 (± 1.81)	
Drug free = no/yes	54/25	31/21	16/1	7 /3	
Inpatient/outpatient	60/19	36/16	14/3	10/0	
Mean number of hospitalization	1.27 (± 0.74)	1.25 (± 0.80)	1.36 (± 0.63)	1.20 (± 0.63)	
Family psychiatric disorder = yes/no	50/29	37/15	9/8	4/6	
Change of diagnosis during study	28	24	4	0	
Change of diagnosis to BP	19	15	4	0	
HAM-D	14.62 (± 8.27)	19.37 (± 5.30)	3.12 (± 2.20)	9.50 (± 3.95)	
YMRS	6.71 (± 9.10)	1.04 (± 1.55)	20.71 (± 6.72)	12.40 (± 5.72)	
S100B (pg/ml) at T0 (median ± SD)	130.1 (± 75.9)	134.6 (± 147.2)	114.6 (± 139.1)	125.1 (± 179.8)	151.9 (± 172.9 )

*HAM-D* Hamilton scale, *YMRS* young scale.

All patients and healthy controls were Caucasian and of Polish origin from the Wielkopolska region in Poland. All recruited patients had follow up visits in the outpatient Department of Psychiatry at the University of Medical Sciences, Poznan. All study participants gave written informed consent. If the patient was under 18 years old, written informed consent was obtained from the parents or legal guardians. The patient inclusion criteria were: age between 12 and 24 years, meeting criteria for clearly demarcated depressive episodes or presence of hypomanic, manic or mixed episodes, inpatients or outpatients. The exclusion criteria were: any severe medical or neurological illness, intellectual disability disorder, a pervasive developmental disorder, pregnancy.

### Study design

In the study we assessed lifetime diagnoses and current mental status examinations. The clinical and biological evaluation was conducted first at the baseline (visit 0), then after reaching a stabilized mood (respectively control visits at 3 or 6 months, after one year, and then two years). The diagnosis was established and verified at each time point of the study by two independent psychiatrists, according to ICD-10 and DSM-IV using: The Kiddie Schedule for Affective Disorders—Present and Lifetime Version (KSADS-PL)^19^ standard instrument to confirm the diagnosis in young patients and the Structured Clinical Interview for DSM-IV (SCID)^20^ in patients 18–24 years of age. During control visits clinical assessment included evaluation of clinical factors, and symptom severity was conducted. Blood samples were also drawn.

### Clinical assessment

The severity of depressive symptoms was determined using the Hamilton Rating Scale (HAM-D-17)^21^, the severity of hypomanic or manic symptoms using the Young Mania Rating Scale (YMARS)^22^. Mood state (euthymic, depressed, or hypo/manic, mixed) was estimated during follow-up evaluations at specific time points: at the baseline (visit 0), at visit K (respectively at 3 or 6 months), after one year (visit 12) and after two years (visit 24). The cutoff points for depressed mood was HAMD ratings ≥ 8, and for hypomanic YMRS ≥ 12. Mood stabilization (euthymia) was defined as a mood range when symptoms were subdued enough, so mood and patient activity were not significantly affected. Mood stabilization (euthymia) is defined in researches based on concurrent depression/mania scores on mood rating scales Hamilton (HAMD) and Young scale (YAMRS). The current symptoms' cutoff scores in clinical scales to define euthymia were YMRS score ≤ 12 and HAMD scores ≤ 7. Depending on presented symptoms (depression, hypomania/mania), all patients after baseline had adequate pharmacological treatment with antidepressants: selective serotonin reuptake inhibitors (SSRI), tricyclic antidepressants (TCA) or mood stabilizers respectively. Patients had pharmacological treatment throughout the study depending on the severity of mood symptoms.

### S100B ELISA determination

10 ml of venous blood was drawn into anticoagulant-free tubes between 07:30 and 09:30 after overnight fasting. After 1 h incubation, the serum was separated by centrifugation, aliquoted, and stored at − 70 until analyses the entire sample size could be achieved. The S100B concentration was measured in all samples. Enzyme-linked immunosorbent assay analyses were performed using DuoSet (cat. No DY 1820-05) ELISA Development Kit (R&D System, Minneapolis, MN, USA) according to the manufacturer's instructions, with minor modifications. Plates were coated with the capture antibody (4 µg/ml in PBS) overnight at 4 °C, then washed (3 times) and blocked for three hours in reagent diluent (1% Bovine Serum Albumin (BSA)/Phosphate Buffered Saline (PBS). Serum samples were diluted 1:2 in reagent diluent to avoid any matrix effect. Plates were incubated with 100 μL of samples or standards overnight at RT. All samples and standards were run in duplicates. Detection steps were performed strictly according to the manufacturer’s instructions. All plates were run in one batch, on the same kit lot#, by the same experienced operator. Standard curves ranged from 1500 to 23.4 pg/ml. Intra-assay and inter-assay variability was < 5% and < 10% coefficient of variation (CV) (accordingly).

### Statistical analyses

The Kolmogorov-Smirnoff test was used to check the normality of the data. S100B levels showed non-normal distribution, thus nonparametric methods were used. The U-Mann–Whitney test, Kruskal–Wallis Anova, and paired Wilcoxon signed rank test were applied in the analyses. Linear regression was used in S100B x symptoms severity, and in gender analyses. Spearman’s rank correlation between S100B baseline levels and clinical variables was performed. The significance level after Bonferroni correction for multiple testing was set at p < 0.007. The statistical analyses were performed using the R project^23^ and Statistica v13 software^24^.

### Ethics approval

The Ethics Committee approved the study protocol at the University of Medical Sciences in Poznan (no. 362/11). All experiments were performed in accordance with relevant guidelines and regulations.

### Consent to participate

Written informed consent was obtained from all individual participants included in the study. Informed consent was obtained from parents/legal guardians.

## Results

At baseline 79 patients were included in the study: 52 patients with depression, 17 patients with hypo/mania, and 10 patients with mixed affective disorder diagnosed according to DSM-IV criteria. During follow-up visits, 38 patients with depression and 10 patients with hypo/mania achieved full remission (euthymia) in this group, whilst 5 patients did not fulfill the remission criteria during the study period. Patients with mixed bipolar disorder who did not reached euthymia were excluded from statistical analyses (n = 15). The drop-out rate during the two year observation was 25%. The majority of the reasons for drop out was a lack of compliance. During a 2-year observation in the study, 19 patients changed diagnosis from unipolar to bipolar disorder. There was a significant improvement in HDRS-17 (p < 0.01) or YMRS (p < 0.01) scores during treatment in the depression subgroup, as well as in the hypomanic subgroup. The clinical characteristics are presented in Table 1.

### Baseline comparisons

Decreased S100B levels in the whole group of patients at baseline were detected, compared with healthy controls (p = 0.03). Lower S100B levels in the depressed (p = 0.06) and hypo/manic patients (p = 0.03) compared to controls was found, though after correction for multiple testing differences were not significant. There were no differences in S100B serum concentrations between depression and hypo/manic patients subgroups (p = 0.34). The S100B levels were not related to the severity of depression (HAMD scores) (p = 0.53**)** or hypo/manic (YMRS scores) (p = 0.34) symptoms.

**Figure 1:**
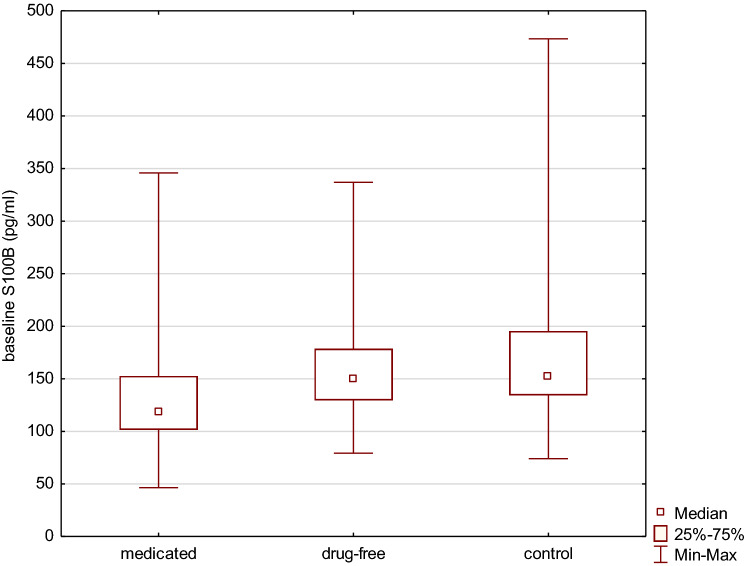
Comparison of baseline S100B serum levels between drug-free, medicated patients and controls. Medicated *vs.* control p = 0.007; medicated *vs.* drug free p = 0.04; drug-free vs. control p = 0.55. Graph was generated using Statistica v13 software.

In the analysis with regard to baseline medication status, drug-free patients had higher S100B levels compared to medicated participants (p = 0.04). Comparing with healthy controls medicated patients had significantly lower S100B levels (p = 0.007), while no differences in S100B levels was found in drug-free group (p = 0.55), Fig. 1.

Dividing patients with regard to family history of psychiatric disorders and, in particular, affective disorders, we found higher S100B levels in depressed patients with family occurrence of affective disorders (p = 0.03), Table 2. Severity of depression at baseline of patients with family history of affective disorders was not different compared to patients with no family loading with affective disorders (p = 0.76).

**Table 2:** Family history of psychiatric and affective disorders.

Group	Family history of psychiatric disorders	Family history of affective disorders
Whole group	p = 0.8	p = 0.1
Depressed	p = 0.19	**p = 0.03**
Hypo/manic	p = 0.32	p = 0.36

Analyzing patients by gender, no significant differences appeared in S100B levels between males and females in the patient group (p = 0.09) or in the control group (p = 0.65). Female control participants had higher S100B levels compared with female patients (p = 0.006). This difference was more significant in medicated females (p = 0.002). We did not observe differences in the male study and control groups (p = 0.9).

There were no differences in baseline S100B concentrations in patients who switched diagnosis from unipolar to bipolar disorder during the 2-year observation period, compared with those without diagnosis change (p = 0.52).

Raw results of baseline comparisons are presented in Table 3.

**Table 3:** Baseline S100B levels comparisons with regard to clinical variables.

Clinical variable	U	Z	p
Depression vs. hypo/manic	322.0000	−0.953517	0.3403
Depression vs. control	559.5000	−1.84740	0.0647
Hypo/manic vs. control	152.0000	−2.14.404	**0.0320**
Diagnosis change	97.00000	−0.622171	0.5338
Drug-free patients vs. medicated patients	308.0000	−2.04468	**0.0409**
Drug-free vs. control	293.0000	−0.596777	0.5507
Medicated vs. control	418.5000	−2.71157	0.0067*
Family history of psychiatric disorders	423.0000	0.238988	0.8111
Family history of affective disorders	387.0000	1.648048	0.0993
Females vs. males study group	315.0000	1.645769	0.0998
Females vs. males control group	56.00000	−0.456523	0.6480
Females study group vs. females control group	355.0000	2.739139	0.0062*
Males study group vs. males control group	46.00000	0.071082	0.9433

Mann–Whitney U-test results.

*Significant after Bonferroni correction for multiple testing.

### Longitudinal comparisons

While comparing S100B concentrations in depressed patients at baseline and after reaching an euthymic state we did not find differences (p = 0.56). Furthermore, neither did we find any such differences in hypo/manic patients (p = 0.2).

There were no differences between baseline and the 2-year visit in S100B levels in patients who completed the follow-up (p = 0.07).

### Correlation analysis of baseline S100B levels with clinical factors

A Spearman’s correlation analysis showed a weak negative correlation of S100B levels with age (R = − 0.253; p = 0.04) and a positive correlation with medication status (R = 0.258; p = 0.04) in the whole study group. A positive correlation with family history of affective disorder in the depressed patients was found (R = 0.321; p = 0.03), Table 4.

**Table 4:** Spearman’s correlations of baseline S100B levels with clinical factors in the patients group.

	Whole group		Depressed		Hypo/manic	
	R	p	R	p	R	p
HAMD scores (baseline)	0.032	0.8	−0.077	0.6	−0.269	0.31
YMRS scores (baseline)	−0.237	0.06	−0.234	0.11	−0.316	0.23
Age	**−0.253**	**0.04**	−0.270	0.06	−0.100	0.71
Gender	−0.208	0.1	−0.239	0.1	−0.150	0.58
Medication status at baseline (drug free vs. medicated)	**0.258**	**0.04**	0.250	0.09	0.252	0.35
Diagnosis change	0.008	0.95	−0.012	0.93	−0.125	0.64
Family history of psychiatric disorders	−0.031	0.81	0.052	0.72	−0.271	0.31
Family history of affective disorders	0.208	0.1	**0.321**	**0.03**	-0.248	0.35

In the control group no correlations with age (R = -0.13; p = 0.49) or gender (R = 0.08; p = 0.64) were found.

## Discussion

Alterations in the S100B level may play a role in various diseases of the central nervous system, including mood disorder^25–27^. Previous studies in adults show elevated S100B serum levels in patients with mood disorders (both depression and mania) compared to the control group. This has been confirmed by a recent meta-analyses^28,29^.

Elevated S100B CSF/serum levels are consistently reported as a state marker in unmedicated patients with major depressive disorder (MDD). Some studies show a correlation with symptom severity. In longitudinal studies MDD patients with higher baseline S100B levels exhibit a better response to antidepressant treatment. An elevated S100B level is detected not only in psychiatric conditions but also in many other neurological disorders such as traumatic brain injury, malignant melanoma, amyotrophic lateral sclerosis, and subarachnoid hemorrhage^17,30^.

A study by Arora et al. (2019) on adolescent Indian major depressive disorder patients detected higher levels of S100B in female patients than in controls, and elevated S100B in recurrent episodes compared to first-episode and controls were reported^31^. The second study on adolescent groups (psychotic and with mood disorders) presents a significant elevation of S100B in patients with childhood trauma, compared to patients without trauma as well as controls. Medication status did not influence on S100B concentration. The study was conducted on a younger group than the one in our experiment. Moreover, earlier age at onset and more prevalent medication indicate more severe symptoms, and fewer unmedicated patients compared to our group makes the results incomparable^32^. In the most recent study by Ottesen et al. (2020) on a large group of monozygotoic twins with (n = 115), at risk (n = 49) or without (n = 40) affective disorders, no differences in S100B levels were noticed^33^.

Decreased baseline S100B levels in patients group might be a result of medication used before entry in the presented study and is gender-dependent. The difference in S100B levels is most significant in medicated female patients compared to healthy female controls. S100B levels in drug-free patients are comparable to controls.

The effect of medication on S100B levels is not well studied. A recent study by Hidese et al. (2020) comparing S100B levels in cerebrospinal fluid of schizophrenic, bipolar disorder, and major depressive disorder patients detected significantly lower S100B concentrations in treated patients in comparison with drug-free patients only with bipolar disorder^34^. The effect of medication on peripheral S100B levels in clinical groups was studied, with contradictory results. Some show no difference during treatment^35–38^ while others report a decrease in S100B concentration. Ginko biloba extract in conjunction with escitalopram as well as with escitalopram alone lowered S100B in elderly patients with depression^39^. Furthermore, study by Schroeter et al. (2002) shows that antidepressant treatment reduces S100B levels in mania and depression^40^. Although citalopram and reboxetine was shown to decrease S100B levels in depression during treatment, baseline and post-treatment S100B levels were higher than controls^36^.

In animal models, fluoxetine increase S100B levels in a rat postnatal brain development^41^. In the rat depression model, chronic unpredictable mild stress significantly increases S100B expression (on mRNA and protein level) in the hippocampus. Venlafaxine administration restores basal S100B levels^42^.

A possible explanation of the observed differences between the adult and adolescent populations may be the heterogeneity of neurotrophic activity in younger patients and compensatory mechanisms in this age group. Glial changes are a dynamic process depending on several factors. During the adolescent period, a spontaneous process of modulation and synaptogenesis is observed, associated with the central nervous system's maturation. Higher levels of serum S100B may be related to neuronal growth and synaptogenesis during synaptic reconstruction in patients with depression^43^. In the meta-analysis, S100B levels were significantly higher in older patients than in younger patients. It is still unclear whether the different S100B protein levels observed in patients with mood disorders in different ages are associated with the progression of the disease or compensatory mechanisms. The results obtained from previous studies may suggest that S100B may be an age-dependent biomarker with an increasing trend during life, but more studies are needed to determine S100B differences between a child, adolescents, and adult patients^44^.

Although we did not find significant differences in S100B levels between the male and female groups, we observed some significant differences in the female subgroup. Lower S100B levels in depressed females compared to healthy female controls were observed, both at baseline and after reaching an euthymic mood.

Our results are in contrast with studies where S100B was significantly elevated in female patients with major depression compared to controls^31,45^. Some studies reported that gender did not have a significant impact on S100B levels^35,36,46,47^. On the contrary, Nygaard et al. (1997) found significantly higher S100B concentrations in CSF in men than in women^48^. Gender differences may be associated with hormonal changes during puberty, indicating complex interactions between biological factors at this age. It will be important to examine the effect of gender on serum S100B levels in a larger group of young patients with mood disorders in the future.

In our study S100B levels were not significantly related to the severity of depression or hypo/manic symptoms, measured using HAMD-17 or YMRS scales, respectively. No significant differences were observed after reaching an euthymic mood. Arora et al., in the study of child and depressed adolescent patients, did not find a correlation between S100B levels and clinical symptoms severity, as assessed by the Beck scale, either^31^. No significant correlations between S100B levels and severity of affective symptoms were found in the most studies on adult groups, for a review see Kroksmark and Vinberg (2018)^29^. Studies which assessed the impact of the severity of depression symptoms and the effects of antidepressant treatment showed a statistically significant correlation between the obtained changes in the Hamilton Depression Rating Scale (HDRS) and changes in the serum s100B level during pharmacotherapy. Patients who responded better to antidepressant treatment had a higher S100B serum level at the baseline, compared to those with a worse response at treatment, which may be associated with the influence of elevated S100B protein levels on plasticity processes in CSN^40,49^. However, this hypothesis requires further research due to the lack of comparable studies on a large group, especially young patients. The authors postulate a possible role of s100B protein in the assessment effectiveness of antidepressant treatment and achieving an euthymic mood.

Positive correlation between S100B levels and family history of mental disorders was reported by Yang et al. (2008)^45^. In our study, a correlation with family history was detected as more specific, in the group with depressive episodes and a family history of affective disorder. In both studies higher S100B was correlated with family loading, which may suggest stronger a genetic influence on the control of S100B expression.

## Conclusions

The results of our study indicate that S100B might be a trait-dependent rather than a state-dependent marker. Due to the lack of similar studies in the youth population, further research should focus on determining whether the differences in S100B levels in specific mood episodes reflect compensatory mechanisms or are instead an expression of glial pathology. In the light of the results obtained, it is worth considering the gender and age of patients and the degree of response to drugs, especially in the drug-free population. Our research results did not confirm the results observed in adult patients; therefore, they are worth pursuing with a larger group of patients.

### Limitations

We would like to point out the most important limitations in our work. These are a relatively small sample size, a lack of an exact age-matched control group, the drop-out rate. We are aware that such exploratory research cannot have evidential value. We have hope that this study may pave the way for future studies with larger samples. For further studies, adolescents and young patients should be followed-up with a more extended period to monitor possible changes in the S100B serum related to age. An interesting issue worth pursuing in future research is diagnosis change from unipolar to bipolar affective disorder in youths and its correlation with the S100B serum.

## Data Availability

Data are available on request.
